# Alveolar capillary dysplasia with misalignment of pulmonary veins in a premature newborn: the role of lung ultrasound

**DOI:** 10.1186/s13089-023-00310-z

**Published:** 2023-02-11

**Authors:** Macarena L. Atun, Silvia A. Fernandez Jonusas, Cecilia M. Acosta

**Affiliations:** 1 Department of Neonatology, Hospital Italiano of Buenos Aires, Buenos Aires, Argentina; 2 Department of Anesthesia, Hospital Privado de Comunidad, Córdoba 4545, 7600 Mar del Plata, Buenos Aires Argentina

**Keywords:** Congenital alveolar dysplasia, Neonate, Pulmonary hypertension, Respiratory distress, Lung ultrasound

## Abstract

**Background:**

Alveolar capillary dysplasia with misalignment of pulmonary veins (ACD/MPV) is a lethal neonatal lung disorder characterized by the decrease of the alveolar units, abnormalities in the air–blood barrier of the lung, and impaired gas exchange. Typically, it affects a full-term newborn; the symptoms usually start within a few hours after birth, resulting in severe respiratory distress and pulmonary hypertension. In most of the cases, this disorder is refractory to conventional pulmonary support.

**Case presentation:**

We report a case of a newborn male of 29 weeks gestational age, with birth weight of 850 g and intrauterine growth restriction. Severe respiratory distress appeared a few minutes after birth; non-invasive ventilatory support was provided in the delivery room and, as a consequence of persistent respiratory failure, he was admitted to the neonatal intensive care unit (NICU) where mechanical ventilation was required.

Due to the symptoms and pulmonary ultrasound pattern suggestive of respiratory distress syndrome, surfactant treatment was administered. Lung ultrasound (LU) was used for monitoring the responsiveness to surfactant; severe pulmonary hypertension ensued, followed by respiratory failure, refractory shock, and death within 48 h.

Owing to the poor response to the established therapy, ACD/MPV was suspected. The diagnosis was confirmed through autopsy. The main goal of this case report is to show the role of LU for monitoring the evolution of this disorder.

**Conclusion:**

LU could provide essential information to help diagnose and follow-up the underlying cause of persistent pulmonary hypertension of the newborn in an earlier and more effective way than chest X-ray. LU is suitable for routine monitoring of lung disease in the NICU.

**Supplementary Information:**

The online version contains supplementary material available at 10.1186/s13089-023-00310-z.

## Background

Alveolar capillary dysplasia with misalignment of pulmonary veins (ACD/MPV) is an uncommon and lethal neonatal lung disorder caused by abnormalities in the air–blood barrier of the lung, decrease in the number of functional air–blood barriers, and impaired gas exchange [1, 2]. It is characterized by severe respiratory failure and persistent pulmonary hypertension of the newborn (PPHN) leading to progressive respiratory failure refractory to all standard medical therapies in most of the cases [1–3]. Most patients develop symptoms within the first 24 h of life and mortality is almost 100% [3].

## Case presentation

We report a case of a preterm male baby, born at 29 weeks of gestational age gestational age (GA), with birth weight of 850 g, Apgar test of 6/8, and intrauterine growth restriction; including a perinatal history of a 44-year-old mother with in vitro fertilization (donation of both gametes), SARS-CoV-2 in the 1st trimester, pre-eclampsia. He received pulmonary maturation with 2 doses of prenatal steroids, and intravenous magnesium sulphate infusion as a neuroprotector. Cesarean section was performed due to Doppler alteration (reverse flow in the umbilical arteries) and decreased fetal movements.

**Fig. 1:**
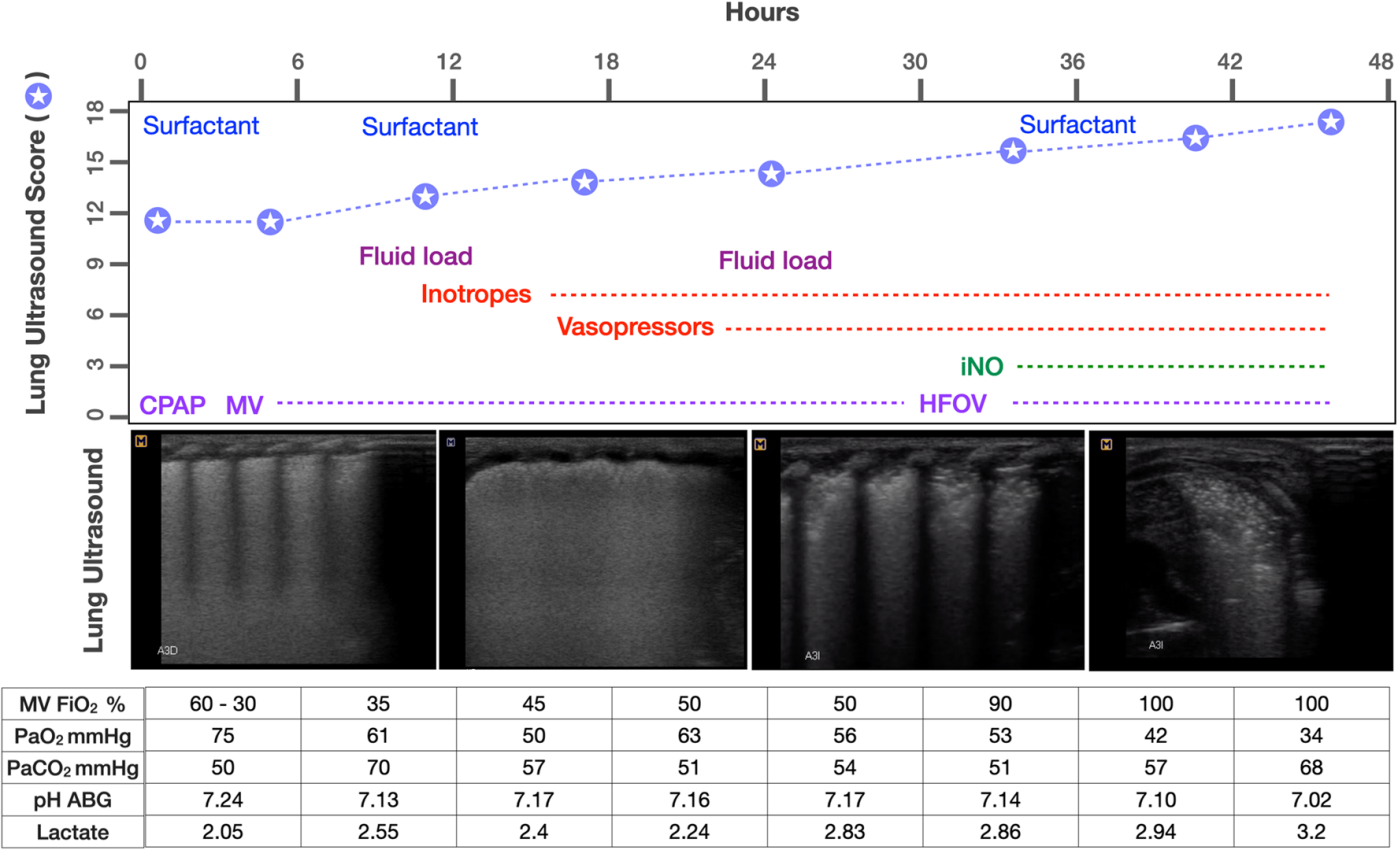
Medical therapy, LU score trend and clinical parameters during the first 48 h of life. LU pattern representatives for each time. *ABGs* arterial blood gases, *iNO* nitric oxide, *CPAP* continuous positive airway pressure, *MV* mechanical ventilation, *HFOV* high-frequency oscillation ventilation

**Fig. 2:**
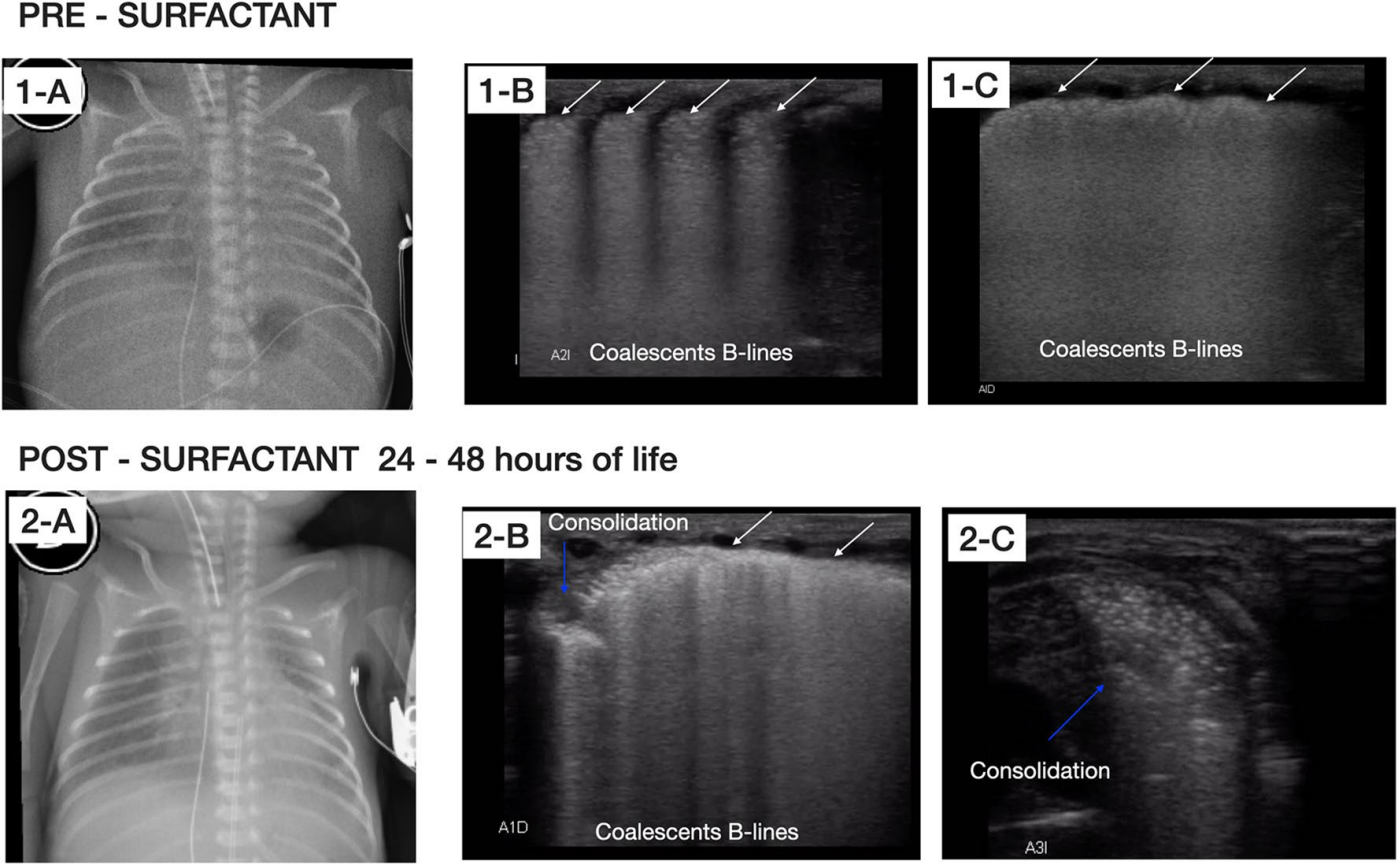
Radiographic and LU examination pre-surfactant administration and after 24–48 h of life. Top: chest X-ray and LU examination after birth pre-surfactant administration. **1-A** Chest X-ray shows poorly inflated lungs with ground glass shadowing widespread in both hemithorax with obliteration of cardiac silhouette. **1-B** and **1-C** LU examinations in longitudinal and oblique view, respectively, show irregularity of the pleural line (white arrows), subpleural consolidation and widespread appearance of “white lung” on both lung fields without spared areas. Bottom: chest X-ray and LU examinations performed after 24–48 h of life. **2-A** chest X-ray shows increased interstitial infiltrate. **2-B** and **2-C** LU examinations in oblique view, show severe deterioration of lung aeration, presence of multiple bilateral consolidations (blue arrows), air bronchograms, and coalescent B-lines without spared areas

Severe respiratory distress appeared within minutes after birth; non-invasive ventilatory support was provided in the delivery room (Fig. 1). Because of persistent respiratory failure, endotracheal intubation and mechanical ventilation were required with high FiO_2_ (60%) and he was admitted to the neonatal intensive care unit (NICU). Chest X-ray showed poorly inflated lungs with ground glass shadowing widespread in both hemithorax with obliteration of cardiac silhouette. At the same time, lung ultrasound (LU) was also performed on admission to the NICU using a high-frequency linear probe of 5–12 MHz (Samsung-Medison MySono U6), revealing thickening and irregularity of the pleural line, subpleural consolidation with tidal recruitment and widespread appearance of white lung (coalescent B-lines) on both lung fields without spared areas (Fig. 2), (Additional file 1: Video S1), LU score of 12 using the LU examination method described by Brat et al. [4]. Therefore, a first dose of Poractant alfa surfactant (CUROSURF) at 200 mg/kg was administered.

After a brief compensation, arterial blood gasses (ABGs) showed progressive impaired oxygenation and ventilation, which required a progressive increase in mechanical ventilation parameters. Oxygenation index progressed from 16 to 50, and LU score showed scarce improvement after the first dose of surfactant, so a second dose at 100 mg/kg was administered.

Echocardiography was performed, ruling out structural heart disease and confirming moderate pulmonary hypertension. Abdominal renal ultrasound revealed bilateral cortical cysts with normal parenchyma and urinary tract characteristics. At 18 h of life, he presented with arterial hypotension, predominantly diastolic, requiring expansion with normal saline, high doses of vasopressors and inotropic agents.

One day later, guided by the ABGs, the mechanical ventilation was set higher, without improvement in the respiratory condition. For this reason, ventilation by high-frequency oscillation (HFOV) was started and, due to PPHN, inhaled nitric oxide at 20 ppm was administered, without response. A new chest X-ray was obtained, showing an increased interstitial infiltrate. Coincidentally, further examination with LU showed severe deterioration of lung aeration, presence of multiple bilateral consolidations, and coalescent B-lines without spared areas suggesting the possibility of respiratory distress syndrome (RDS) with non-response of surfactant therapy (Fig. 2), (Additional file 2: Video S2). The baby received an additional third dose of the same surfactant at 100 mg/kg.

Due to the severity of the clinical picture and increased C-reactive protein, retro cultures were taken from arterial and venous catheters and the baby was empirically medicated with ampicillin–gentamicin. In the presence of the worsening of the clinical picture and increased C-reactive protein, the antibiotics were shifted to vancomycin–amikacin. All cultures were negative.

The patient developed hypotension refractory to medical treatment, such as fluid expansion, inotropes, high doses of vasopressors, and systemic corticosteroids. Also associated with deterioration of the clinical picture, there was a generalized edema and decreased diuretic rhythm with worsening of the renal function. Compared to admission, LU showed an increased pattern of alveolar interstitial syndrome, with no response to surfactant therapy.

On day 2, he developed persistent hypoxemia with severe pulmonary hypertension, saturation 50%, PO_2_ 34, OI 50, with a FiO_2_ of 100% and sustained arterial hypotension despite the established treatment, followed by pulmonary hemorrhage and cardiorespiratory arrest with no response to resuscitation maneuvers.

**Fig. 3:**
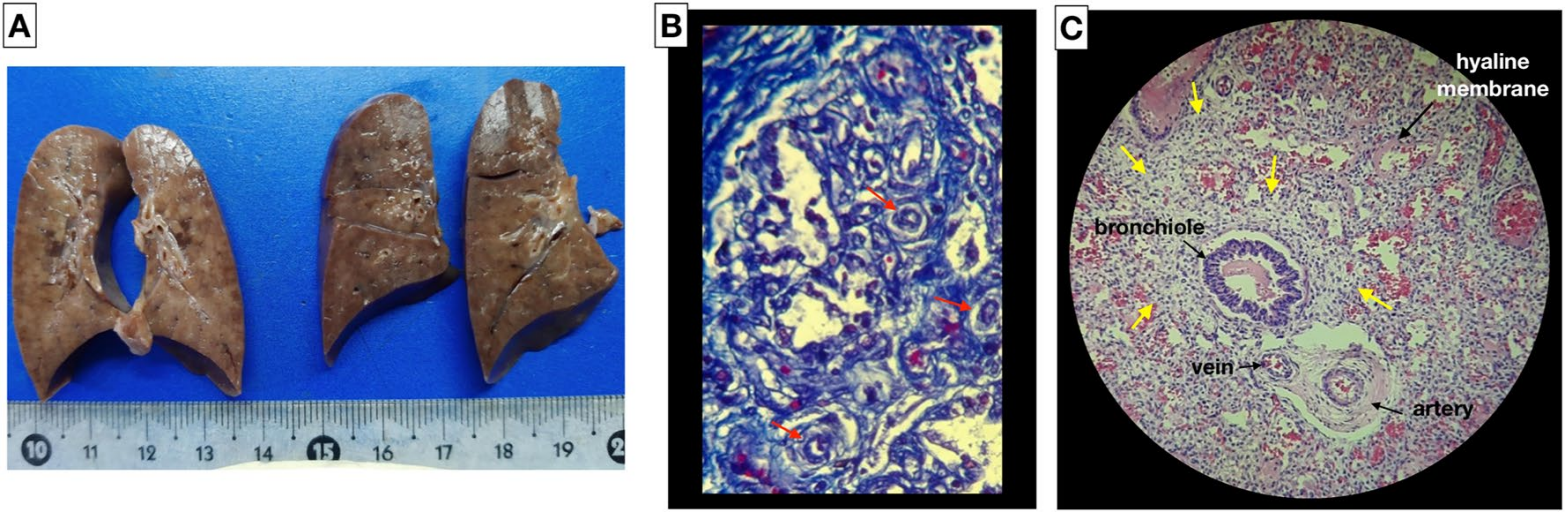
Macroscopic and histopathological finding of alveolar capillary dysplasia with ACD/MPV. **A** Macroscopic image and the cut surface of the lungs. Histopathology study: **B** abnormal capillary pattern (red arrows) in the pulmonary interstitial space (Masson’s trichrome stain); **C** thickening of the alveolar wall (yellow arrows), irregular capillary vascularization, hypertrophy of the medial layer of the arterioles, abnormal arrangement of the veins adjacent to the arteries and foci of hyaline membranes (hematoxylin and eosin stain)

ACD/MPV was suspected due to the poor response to the established therapy, confirming the diagnosis by autopsy, which shows poorly developed lung parenchyma for GA with thickening of the septa, irregular capillary vascularization, hypertrophy of the medial layer of the arterioles, abnormal arrangement of the veins adjacent to the arteries and the presence of lymphangiectasia. Foci of hyaline membranes and foci of alveolar hemorrhage were also recognized in patches in both lungs, which is compatible with alveolar capillary dysplasia with misalignment of the pulmonary veins (Fig. 3). The autopsy corresponds with the outcome and lack of response to the established therapy.

## Discussion

Infants affected with ACD/MPV have progressive respiratory difficulties within the first hours of life and develop respiratory distress and refractory pulmonary hypertension [1, 2]. Although there are reports indicating that more than 90% of the affected newborns are born full term with normal weight and Apgar scores [2], in this case the neonate was a preterm of 29 weeks GA. The clinical presentation also depends on associated congenital malformation because more than 80% of infants may have other malformations affecting the cardiac, gastrointestinal, genitourinary, and musculoskeletal systems.

Within the complementary studies, the chest X-ray films show non-specific signs, such as bilateral opacity or pneumothorax; CT scans show a ground glass image with thickening of the septal line, vascular alterations are not appreciated since the peripheral capillaries of the lung are not visualized. On the other hand, echocardiography allows for confirmation of pulmonary hypertension and rules out structural cardiovascular abnormalities [5]. However, to our knowledge, the usefulness of LU in the follow-up of a patient with ACD/MPV has not been reported before in preterm babies.

Respiratory distress is the most common respiratory neonatal disease. In recent years, LU has been gaining consensus as a safe, non-invasive, bedside, radiation-free tool for the diagnosis and follow-up of several lung conditions in neonatal and pediatric patients [6, 7]. The typical ultrasound findings of SDR are the irregularity of the pleural line with subpleural consolidation and bilateral widespread appearance of “white lung” without spared areas [7]. Many studies have described the usefulness of LU in the diagnosis of RDS and highlight the use of this imaging tool in the assessment and follow-up of a neonate with respiratory distress [7–9]. In a recent review, Corsini et al. reported that LU has a sensitivity of 96.7% and a specificity of 100% for the diagnosis of RDS in neonates. Moreover, considering chest X-ray as a gold standard, they found an excellent agreement of 96.7% [6].

However, it should be kept in mind that the combination of LU, chest X-ray, clinical picture and laboratory data must always be integrated to improve the diagnosis of rare conditions of respiratory distress in newborns, such as congenital emphysema, congenital *Ureaplasma* spp. infection, and some specific pulmonary malformations.

In our preterm neonate, due to the of onset severe respiratory distress within minutes after birth, LU examination was performed. Although LU shows non-specific sonographic signs of ACD/MPV, this non-invasive tool has a crucial advantage, since it allows to assess the severity of respiratory distress, as well as to monitor the progression of lung disease, quantifying changes in lung aeration with high sensitivity.

Nowadays, LU has also been used as a semiquantitative method to monitor the progress of a disease and to decide whether to perform a specific treatment, which was defined by Raimondi et al. as a “functional tool” [9]. Consequently, semiquantitative LU score has been applied in neonatology to predict the need for exogenous surfactant therapy [4, 10–13]. The predictive accuracy of LU score in neonates was better in preterm infants with GA of less than or equal to 30 weeks, as shown in the area under the curve (AUC) of the ROC curve of 0.94 (95% CI 0.90–0.98). In a recent study, Perri et al. showed that preterm infants with RDS and with LU score ≥ 7 obtained 2 h after exogenous surfactant administration can be useful in identifying patients who will need a second course, showing a sensitivity and specificity of 94% and 60%, respectively [12].

Furthermore, the LU score was shown to predict the need for exogenous surfactant more accurately than the radiological score, with a sensibility of 86% vs. 82%, and a specificity of 88% vs. 76%, respectively [13]. The aim is not to rule out conventional chest X-ray in the management and follow-up of neonates with respiratory distress, but rather to reduce its use and avoid unnecessary exposure to ionizing radiations [8, 9].

The mortality of ACD/MPV is almost 100% within the first month of life, despite using supportive care, including extracorporeal membrane oxygenation [1–3, 5]. In our patient, ACD/MPV was suspected due to the lack of response to all established therapy, with severe impairment of lung aeration, as shown by LU (Fig. 1) and chest X-ray (Fig. 2).

A final diagnosis of ACD/MPV is made by pathological examination through lung biopsy or lung tissue autopsy. However, the incidence of the disease is unknown, as many cases pass undiagnosed. Recently, Deng et al. reported a non-invasive method such as DNA sequencing and FOXF1 analysis that could be helpful in the clinical diagnosis of ACD/MPV [14].

## Conclusion

In a preterm neonate, the presence of RDS with PPHN refractory to established medical therapies, the presence of ACD/MVP should be suspected.

LU has become an extremely useful imaging tool in neonates with RDS in the NICU, allowing not only a diagnosis but also the monitoring of the progression of the disease through a semiquantitative method that helps decision-making as to which treatment to follow.

## Supplementary Information

**Additional file 1: Video S1.** LU videos performed on admission to the NICU in longitudinal and oblique view showing subpleural consolidation with tidal recruitment and widespread appearance of white lung on both lung fields without spared areas.

**Additional file 2: Video S2.** Top: LU videos performed at 30 h of life in longitudinal and oblique view showing severe deterioration of lung aeration, presence of multiple bilateral large consolidations, and coalescent B-lines without spared areas. Bottom: Transthoracic echocardiography: apical four-chamber view, note the abnormal septal motion; pulmonary artery systolic pressure was measured with trans-tricuspid continuous wave Doppler as equal to 61 mm Hg (49 mm Hg trans-valvular gradient + 12 mm Hg central venous pressure).

## Data Availability

The data used in the present case report are available from the corresponding author on reasonable request.

## Abbreviations

ACD/MPV: Alveolar capillary dysplasia with misalignment of pulmonary veins
NICU: Neonatal intensive care unit
RDS: Respiratory distress syndrome
LU: Lung ultrasound
PPHN: Persistent pulmonary hypertension of the newborn
GA: Gestational age
ABGs: Arterial blood gases
HFOV: High-frequency oscillation
iNO: Inhaled nitric oxide
CPAP: Continuous positive airway pressure
MV: Mechanical ventilation
